# Serum small dense LDL cholesterol is inversely associated with kidney stone prevalence: evidence from two independent cross-sectional studies

**DOI:** 10.3389/fendo.2026.1816344

**Published:** 2026-04-29

**Authors:** Yangyang Zhang, Haibo Qin, Junping Yang, Xudong Shen, Zongyao Hao, Chaozhao Liang

**Affiliations:** 1 Department of Urology, The First Affiliated Hospital of Anhui Medical University, Hefei, Anhui, China; 2 Institute of Urology, Anhui Medical University, Hefei, Anhui, China; 3 Anhui Province Key Laboratory of Urological and Andrological Diseases Research and Medical Transformation, Hefei, Anhui, China; 4 Department of General Practice, Wuhu City Second People`s Hospital, Wuhu, Anhui, China

**Keywords:** Dyslipidemia, kidney stones, metabolic risk factors, small dense low-density lipoprotein cholesterol (sdLDL-C), urolithiasis

## Abstract

This study investigated the association between serum small dense low-density lipoprotein cholesterol (sdLDL-C) levels and kidney stone formation in adults using two independent datasets. A hospital-based cross-sectional cohort included 757 participants (229 stone cases and 528 controls) from March 2022 to September 2025, with clinical data on blood biochemical markers and histories of hypertension and diabetes collected for analysis. Between-group comparisons were conducted using chi-square or Kruskal–Wallis tests, and the association between sdLDL-C and kidney stones was examined through multivariable logistic regression, subgroup analyzes. To evaluate the robustness and generalizability of the findings, data from 9,721 adults in the 2007–2016 National Health and Nutrition Examination Survey (NHANES) were also analyzed. In the Chinese cohort, higher sdLDL-C concentrations were independently associated with a lower risk of kidney stones (OR = 0.53, 95% CI: 0.38–0.74), with a non-linear inverse dose–response pattern. The NHANES cohort showed similar results, revealing a negative and linear association between sdLDL-C and stone risk (OR = 0.68, 95% CI: 0.50–0.91). Subgroup analyzes indicated that the protective association was most evident among individuals over 40 years and in both sexes in the Chinese cohort, whereas in NHANES it was more pronounced among participants aged 20–39 or 60–85 years and among females. These findings suggest that sdLDL-C is inversely associated with kidney stone risk across populations; however, prospective studies are required to determine causality.

## 1 Introduction

Kidney stones are one of the most common urinary system disorders, characterized by an imbalance between urinary crystals and colloids, resulting in abnormal mineral salt deposition in the kidneys and subsequent stone formation (1). The global burden of kidney stone disease is increasing, with epidemiological surveys from developed countries reporting prevalences ranging from 5% to 15% (2). As a populous country, China has also experienced a significant rise in kidney stone incidence. A recent nationwide cross-sectional study reported a kidney stone prevalence of 6.4% among Chinese adults, with the onset age continuously decreasing and an increasing proportion of young and middle-aged patients annually (3). A critical clinical feature of this disease is its high recurrence rate; approximately one-third of patients experience recurrent stones after the initial episode (4), with cumulative risk further increasing among those with recurrent histories (5). Systematic reviews suggest a lifetime recurrence rate for kidney stone patients approaching 50% (6). Repeated stone episodes not only cause severe pain, urinary tract obstruction, and renal function impairment but also impose a considerable economic burden. In the United States, annual direct medical costs for kidney stone treatment exceed $5 billion, with significant additional indirect costs related to lost productivity (7, 8). Therefore, identifying effective predictors of recurrence and developing targeted prevention strategies represent critical priorities in urological research.

In recent years, with advances in research, kidney stones have been reconceptualized as a systemic metabolic disease rather than merely a local pathological condition of the urinary system (9). Substantial evidence indicates that kidney stones are closely associated with various chronic diseases, particularly cardiovascular disease, metabolic syndrome, and chronic kidney disease (10, 11). Although the specific mechanisms linking kidney stones with cardiovascular comorbidities remain incompletely elucidated, dyslipidemia is considered a key bridge connecting the two. Multiple studies suggest that dyslipidemia plays a significant role in the formation of kidney stones, partly independent from other components of metabolic syndrome (12–14). Among various lipid indicators, sdLDL-C has garnered significant attention due to its stronger atherogenic potential. SdLDL-C particles, being smaller and denser, can more readily infiltrate the vascular intima and are more susceptible to oxidation. They are closely associated with various metabolic diseases, including type 2 diabetes (15), metabolic syndrome (16), and obesity (17). Nevertheless, the association between sdLDL-C and kidney stone formation remains inadequately defined due to limited and conflicting evidence from prior research.

Against this background, the current investigation utilized two independent cohorts to evaluate the potential relationship between serum sdLDL-C concentrations and kidney stone prevalence. Initially, clinical data from patients diagnosed with kidney stones at our institution were retrospectively analyzed. Subsequently, external validation was conducted using the NHANES dataset from the United States. This approach aimed to ascertain whether sdLDL-C might serve as a valuable biomarker for kidney stone risk prediction and to provide additional epidemiological insights into the metabolic basis of nephrolithiasis.

## 2 Materials and methods

### 2.1 Study population

**Figure 1:**
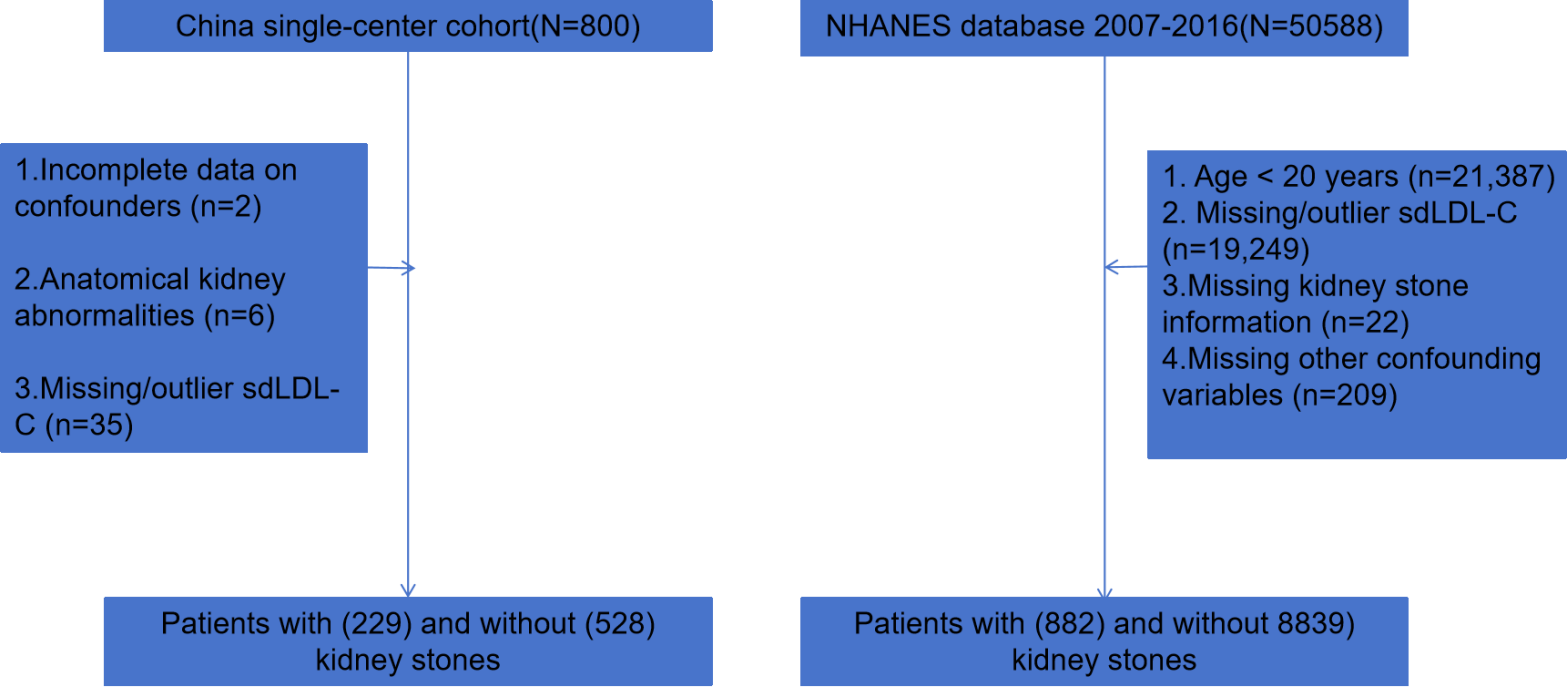
Flowchart of participant selection.

This study received ethical approval from the Ethics Committee of the First Affiliated Hospital of Anhui Medical University and was conducted in accordance with the ethical standards specified in the 1964 Declaration of Helsinki and its subsequent amendments. Clinical data were retrospectively reviewed from 800 patients who underwent surgical intervention for kidney stones in the Department of Urology at the First Affiliated Hospital of Anhui Medical University between March 2022 and September 2025. A concurrent group of healthy controls from physical examination centers was also included. Participants meeting any of the following criteria were excluded (**Figure 1**): (1) incomplete data on confounding factors (n=2); (2) presence of anatomical abnormalities such as solitary kidney or horseshoe kidney (n=6); and (3) missing or outlier sdLDL-C measurements (n =35). Ultimately, 757 participants (229 kidney stone patients and 528 controls) were included in the final analysis.

Concurrently, this study incorporated data from the 2007–2016 US NHANES. The database initially included 50,588 participants. After screening (**Figure 1**), 9,721 participants were ultimately included, among whom 882 reported a history of kidney stones.

### 2.2 Data collection and definitions

Chinese Dataset: Blood samples were analyzed using an automated flow cytometer. Indicators measured included platelet count (PLT), neutrophils (NE), lymphocytes (LYM), urea, total cholesterol (TC), triglycerides (TG), high-density lipoprotein cholesterol (HDL-C), low-density lipoprotein cholesterol (LDL-C), apolipoprotein B (Apo B), serum calcium, creatinine, and blood urea nitrogen. Blood glucose levels were measured with a Roche Cobas 6000 biochemical analyzer using venous samples collected during hospitalization prior to surgery. Additionally, demographic characteristics such as age, gender, and histories of hypertension and hyperglycemia were obtained from past medical records. The exposure variable, sdLDL-C, was calculated according to the formula proposed by Sampson et al. (18): sdLDL-C = 0.14 × ln(TG) × LDL-C − 0.43 × LDL-C + 8.99 (TG and LDL-C in mg/dL).This equation was rigorously validated against the gold-standard ultracentrifugation method and has been widely proven to provide a highly accurate estimation of sdLDL-C, making it a reliable tool for large-scale epidemiological research where direct measurement is unfeasible NHANES Dataset: The NHANES dataset incorporated identical confounding variables to those in the Chinese cohort. TC and TG levels were quantified enzymatically. HDL-C concentrations were determined via the heparin-manganese precipitation assay or direct immunoassay. ApoB was assessed through immunoturbidimetric analysis. LDL-C levels were estimated using the Friedewald formula. All biochemical measurements were conducted using Hitachi 704/717 analyzers or Roche Modular P automated biochemical systems. Importantly, to ensure strict methodological consistency and data comparability between the two independent cohorts, sdLDL-C concentrations in the NHANES dataset were calculated utilizing the exact same Sampson formula applied to the Chinese dataset.

### 2.3 Definition of kidney stones

The definition of kidney stones differed between the two datasets. The Chinese dataset employed computed tomography (CT) or B-ultrasound as diagnostic gold standards, whereas the NHANES dataset relied on self-reported questionnaire data, a method previously validated in numerous studies.

### 2.4 Confounding factors

Multivariable logistic regression analyzes were adjusted for potential confounding factors, including sex (male or female), age (years), body mass index (BMI), hypertension, diabetes, urea, creatinine, total cholesterol (TC), serum calcium, and serum uric acid, to evaluate the association between sdLDL-C and kidney stone occurrence. To control for potential inflammatory effects on kidney stones, the systemic immune-inflammation index (SII) was calculated (SII = peripheral neutrophil count × platelet count/lymphocyte count × 10^9^/L) (19).

### 2.5 Handling of missing data

To address missing values in our datasets, we employed a complete-case analysis approach (listwise deletion). For the Chinese single-center cohort, the proportion of missing data was highly restricted; only 37 out of 800 initially screened individuals (approximately 4.6%) were excluded due to incomplete clinical confounders or unmeasurable sdLDL-C levels. Given that the overall missing rate was strictly below the conventional 5% threshold, the data were considered Missing Completely At Random (MCAR), rendering complete-case analysis statistically adequate without introducing substantial selection bias. Similarly, for the NHANES dataset, the large-scale exclusion of participants was predominantly dictated by the complex survey design (e.g., specific laboratory lipid panels and fasting tests being exclusively administered to predetermined subsamples) rather than patient non-compliance. Consequently, only participants with complete and valid data across all required variables—including self-reported kidney stone history, targeted lipid parameters, and basic demographic profiles—were included in the final regression models to ensure optimal analytical robustness.

### 2.6 Statistical methods

Continuous variables are presented as mean ± standard deviation (SD), and categorical variables as percentages. The Kolmogorov–Smirnov (KS) test was used to assess the normality of continuous variables. For intergroup comparisons, weighted t-tests were used for normally distributed variables, and weighted chi-square tests were applied for categorical variables and non-normally distributed data.

Outliers in sdLDL-C levels were identified as values falling below Q1 – 1.5 × IQR or above Q3 + 1.5 × IQR (where Q1 and Q3 are the first and third quartiles, and IQR is the interquartile range). Participants with such outlying values were excluded from the final analysis to reduce the influence of extreme measurements and enhance the robustness of the statistical findings. To systematically address missing values, we employed a complete-case analysis approach (listwise deletion). Given that the overall missing rate was strictly below the conventional 5% threshold in the Chinese cohort, and massive exclusions in the NHANES dataset were predominantly dictated by the complex survey subsample design, the data were reasonably considered Missing Completely At Random (MCAR), rendering complete-case analysis statistically adequate without introducing substantial selection bias.

Prior to interpreting the multivariable logistic regression models, we rigorously assessed potential multicollinearity among all included covariates by calculating the Variance Inflation Factor (VIF). A VIF value of less than 5 was predefined as an indicator of the absence of significant multicollinearity. Following the recommendations of the STROBE guidelines, three distinct multivariable logistic regression models were constructed to evaluate the independent relationship between serum sdLDL-C levels (analyzed both continuously and divided into tertiles) and kidney stone occurrence. Model 1 represented the crude association without covariate adjustments. Model 2 was adjusted for demographic factors and physical attributes (gender, age, and body mass index [BMI]). Model 3 incorporated additional covariates, including hypertension, diabetes, creatinine, total cholesterol (TC), triglycerides (TG), fasting blood glucose (GLU), uric acid, and serum calcium levels. Furthermore, to minimize the potential bias of reverse causality driven by medication use or diet modifications, a sensitivity analysis was performed by excluding participants with diabetes and hypertension, thereby re-evaluating the core association in the remaining sub-population.

Furthermore, the potential non-linear correlation between sdLDL-C and kidney stone prevalence was examined using generalized additive model (GAM) regression and visualized through penalized spline smoothing. If non-linearity was visually evident, a two-piecewise linear regression model was applied, and a logarithmic likelihood ratio test was subsequently performed to calculate the threshold effect and determine the precise optimal inflection point. Additionally, a linear trend test (P for trend) was conducted by entering the categorical tertile variables as continuous variables in the regression models to confirm the dose-response gradient. Finally, to formally validate the observed heterogeneity across different demographic and clinical subgroups, multiplicative interaction terms (e.g., sdLDL-C × age category, sdLDL-C × gender, sdLDL-C × hypertension, and sdLDL-C × diabetes) were incorporated into the fully adjusted multivariable models (Model 3) to calculate the P for interaction. All statistical analyzes were conducted using Empower software (X&Y Solutions, Inc., Boston, MA, USA; www.empowerstats.com) and R version 4.2.2 (The R Foundation; http://www.R-project.org). Statistical significance was defined as a p-value < 0.05.

## 3 Results

### 3.1 Baseline characteristics of the Chinese population

The Chinese cohort comprised 757 individuals, including 229 with kidney stones (stone group) and 528 without stones (non-stone group). According to **Table 1**, baseline characteristics such as age, BMI, urea, total cholesterol, systemic immune-inflammation index (SII), serum calcium, creatinine levels, gender distribution, and hypertension incidence did not significantly differ between the two groups (all P > 0.05), indicating comparable baseline profiles.

**Table 1:** Baseline data for populations in local regions of China.

Characteristic	Non-stone formers	Stone formers	P-value
N	528	229	
Age(years)	50.50 ± 12.57	51.48 ± 11.95	0.314
BMI(kg/m2)	24.38 ± 3.90	24.32 ± 3.38	0.818
Urea(mg/dl)	16.22 ± 8.96	16.98 ± 7.39	0.272
Serum Uric Acid(mg/dl)	5.49 ± 1.52	5.80 ± 1.66	0.013
Serum Cholesterol(mg/dl)	177.11 ± 36.35	175.95 ± 36.35	0.639
Serum Glucose((mg/dl)	99.00 ± 28.80	93.60 ± 16.20	0.01
SII	533.56 ± 474.02	544.98 ± 815.38	0.81
Serum Calcium(mg/dl)	9.90 ± 0.97	9.42 ± 0.64	0.274
Creatinine(mg/dl)	0.97 ± 1.08	1.07 ± 0.63	0.175
sdLDL-C	4.10 ± 0.48	3.97 ± 0.50	0.001
Gender(%)			0.501
Male	302 (57.20%)	137 (59.83%)	
Female	226 (42.80%)	92 (40.17%)	
High Blood Pressure(%)			0.586
No	356 (67.42%)	159 (69.43%)	
Yes	172 (32.58%)	70 (30.57%)	
Diabetes(%)			0.053
No	431 (81.63%)	200 (87.34%)	
Yes	97 (18.37%)	29 (12.66%)	

Analysis of metabolic indicators showed that serum uric acid was significantly elevated in kidney stone patients compared to controls (5.80 ± 1.66 mg/dL vs. 5.49 ± 1.52 mg/dL; P = 0.013). Conversely, fasting blood glucose (93.60 ± 16.20 mg/dL vs. 99.00 ± 28.80 mg/dL; P = 0.01) and sdLDL-C levels (3.97 ± 0.50 vs. 4.10 ± 0.48; P = 0.001) were significantly lower among stone patients. Additionally, the prevalence of diabetes was marginally higher in kidney stone patients compared to controls (87.34% vs. 81.63%; P = 0.053), although the difference was not statistically significant.

### 3.2 Baseline characteristics of the US NHANES database population

A total of 9,721 participants from the US NHANES database were included, with 8,839 in the non-stone group and 882 in the stone group. Detailed comparisons of baseline characteristics are presented in **Table 2**.

**Table 2:** Baseline population data from the US NHANES database.

Characteristic	Non-stone formers	Stone formers	P-value
N	8839	882	
Age(years)	49.11 ± 17.62	56.88 ± 16.08	<0.001
BMI(kg/m2)	28.87 ± 6.75	30.32 ± 6.64	<0.001
Urea(mg/dl)	13.10 ± 5.85	14.76 ± 6.81	<0.001
Serum Cholesterol(mg/dl)	193.85 ± 41.26	190.73 ± 40.41	0.11
Serum Uric Acid(mg/dl)	5.50 ± 1.42	5.67 ± 1.45	<0.001
Serum Glucose(mg/dl)	102.39 ± 34.69	109.82 ± 39.47	<0.001
Serum Calcium(mg/dl)	9.38 ± 0.34	9.37 ± 0.40	0.722
Creatinine(mg/dl)	0.89 ± 0.42	0.95 ± 0.69	<0.001
SII	518.77 ± 325.45	584.92 ± 997.80	<0.001
sdLDL-C	2.12 ± 0.27	2.06 ± 0.26	0.001
Gender(%)			<0.001
Male	4271 (48.32%)	493 (55.90%)	
Female	4568 (51.68%)	389 (44.10%)	
Diabetes(%)			<0.001
Yes	1007 (11.39%)	185 (20.98%)	
No	7637 (86.40%)	668 (75.74%)	
Borderline	195 (2.21%)	29 (3.29%)	
High Blood Pressure(%)			<0.001
Yes	3128 (35.39%)	452 (51.25%)	
No	5711 (64.61%)	430 (48.75%)	

Further analysis revealed that kidney stone patients were older (56.88 ± 16.08 vs. 49.11 ± 17.62 years; P < 0.001) and exhibited higher BMI values (30.32 ± 6.64 kg/m² vs. 28.87 ± 6.75 kg/m²; P < 0.001) relative to non-stone individuals. Laboratory tests showed significant elevations in urea (14.76 ± 6.81 mmol/L vs. 13.10 ± 5.85 mmol/L; P < 0.001), serum uric acid (5.67 ± 1.45 mg/dL vs. 5.50 ± 1.42 mg/dL; P < 0.001), fasting blood glucose (109.82 ± 39.47 mg/dL vs. 102.39 ± 34.69 mg/dL; P < 0.001), and creatinine levels (0.95 ± 0.69 mg/dL vs. 0.89 ± 0.42 mg/dL; P < 0.001) among stone patients. Additionally, systemic immune-inflammation indices were significantly higher (584.92 ± 997.80 vs. 518.77 ± 325.45; P < 0.001), whereas sdLDL-C concentrations were notably lower in stone patients (2.06 ± 0.26 vs. 2.12 ± 0.27; P = 0.001). No significant differences emerged between groups for total cholesterol or serum calcium.

Regarding demographics and comorbid conditions, male gender prevalence (55.90% vs. 48.32%; P < 0.001), hypertension prevalence (51.25% vs. 35.39%; P < 0.001), and diabetes prevalence (20.98% vs. 11.39%; P < 0.001) were significantly greater among kidney stone patients. In the NHANES cohort, diabetes was further classified into three categories, and intergroup differences remained statistically significant (P < 0.001).

### 3.3 Independent inverse association between sdLDL-C and kidney stone risk validated in two populations

Prior to evaluating the specific associations, multicollinearity diagnostics were performed for the fully adjusted models. The results confirmed that all included covariates exhibited VIF values well below the threshold of 5, indicating no severe multicollinearity within the regression models. To assess the association between serum sdLDL-C and kidney stone risk, three hierarchical multivariable logistic regression models were applied independently to the Chinese and NHANES cohorts (**Table 3**).

**Table 3:** Association between sdLDL-C and kidney stone risk: multivariable logistic regression analysis in Chinese and NHANES populations.

Characteristic	Model 1 OR (95% CI)	Model 2 OR (95% CI)	Model 3 OR (95% CI)	Model 4 OR (95% CI)
China data				
sdLDL-C	0.60 (0.44, 0.82)	0.57 (0.42, 0.79)	0.53 (0.38, 0.74)	0.39(0.25, 0.60)
Categories				
Low (0.092-0.48)	1	1	1	1
Middle (0.48-0.62)	0.84 (0.58, 1.22)	0.82 (0.56, 1.19)	0.78 (0.53, 1.14)	0.73 (0.45, 1.20)
High (0.62-1.85)	0.67 (0.46, 0.99)	0.66 (0.45, 0.97)	0.64 (0.43, 0.95)	0.39 (0.23, 0.68)
P for trend	<0.001	<0.001	<0.001	<0.001
NHANES data				
sdLDL-C	0.43 (0.33, 0.57)	0.65 (0.49, 0.87)	0.68 (0.50, 0.91)	0.75 (0.48, 1.17)
Categories				
Low (0.092-0.48)	1	1	1	1
Middle (0.48-0.62)	0.75 (0.64, 0.88)	0.84 (0.72, 0.99)	0.86 (0.72, 1.01)	0.82 (0.64, 1.06)
High (0.62-1.85)	0.58 (0.49, 0.69)	0.74 (0.61, 0.89)	0.75 (0.62, 0.91)	0.85 (0.64, 1.13)
P for trend	<0.001	<0.001	0.003	0.256

Model 1= No covariates were adjusted.

Model 2= Model 1+ age, gender, BMI.

Model 3 was adjusted for all covariates in **Tables 1**, **2**.

Model 4 was adjusted for the same variables as Model 3 and excluded individuals with hypertension and/or diabetes.

#### 3.3.1 Results from the Chinese cohort

Logistic regression results with kidney stone occurrence as the dependent outcome and sdLDL-C levels as continuous predictors are summarized in **Table 3**. In the crude analysis (Model 1), sdLDL-C demonstrated a significant negative correlation with the risk of kidney stones (OR = 0.60, 95% CI: 0.44–0.82). This inverse association was strengthened in Model 2 (adjusted for sex and age) (OR = 0.57, 95% CI: 0.42–0.79) and remained significant in Model 3 (further adjusted for BMI, hypertension, diabetes, serum uric acid, blood glucose, etc.) and remained significant in Model 3 (further adjusted for BMI, hypertension, diabetes, serum uric acid, blood glucose, etc.) (OR = 0.53, 95% CI: 0.38–0.74). To further test the robustness of our findings against potential reverse causality (e.g., lipid-lowering treatments or dietary modifications common in metabolic diseases), a sensitivity analysis was performed by excluding individuals with hypertension and diabetes (Model 4, **Table 3**). In this relatively healthy sub-population, the independent protective association remained highly robust (OR = 0.39, 95% CI: 0.25–0.60).

Participants were subsequently categorized into three tertile-based groups (low, middle, high) according to sdLDL-C concentrations to further explore this relationship. Compared with the lowest tertile group, the intermediate tertile showed no significant difference in kidney stone risk. However, participants in the highest sdLDL-C tertile had consistently reduced risks across all statistical models. In the fully adjusted analysis (Model 3), individuals in the highest tertile had a 36% lower likelihood of kidney stone occurrence compared to the lowest tertile (OR = 0.64, 95% CI: 0.43–0.95), suggesting that elevated sdLDL-C concentrations might confer protective effects against nephrolithiasis. Furthermore, linear trend tests across the sdLDL-C tertiles revealed a significant dose-response gradient across Models 1 to 3 (P for trend < 0.001, **Table 3**), reinforcing the inverse relationship between sdLDL-C concentrations and nephrolithiasis risk in the Chinese cohort.

#### 3.3.2 Results from the US NHANES database analysis

Similar trends emerged from the NHANES dataset. In unadjusted analyzes (Model 1), sdLDL-C levels exhibited a significant inverse relationship with kidney stone prevalence when analyzed continuously (OR = 0.43, 95% CI: 0.33–0.57). After adjusting for demographic characteristics (sex and age; Model 2) and additional metabolic factors (Model 3), the inverse association persisted, albeit slightly attenuated (Model 3: OR = 0.68, 95% CI: 0.50–0.91).Consistent with the Chinese cohort, a sensitivity analysis excluding patients with hypertension and diabetes was also conducted (Model 4, **Table 3**). While the point estimate continued to suggest a protective trend (OR = 0.75, 95% CI: 0.48–1.17), it lost statistical significance, likely due to the diminished statistical power resulting from the significantly reduced sample size.

Upon stratification into tertiles, the intermediate sdLDL-C group demonstrated significantly reduced risk compared to the lowest tertile only in Model 2 (OR = 0.84, 95% CI: 0.72–0.99); however, this significance was lost following comprehensive adjustment in Model 3. Nevertheless, the highest tertile consistently maintained a significantly reduced risk in the fully adjusted model (Model 3: OR = 0.75, 95% CI: 0.62–0.91).Additionally, the linear trend test confirmed a significant dose-response gradient across Models 1 to 3 (P for trend ≤ 0.003, **Table 3**), indicating that higher sdLDL-C categories are progressively associated with a reduced risk of kidney stones in the US population as well.

### 3.4 Subgroup analysis stratified by age and gender

To further clarify the potential modifying influences of age and sex on the sdLDL-C–kidney stone association, stratified logistic regression analyzes were carried out within both the Chinese and NHANES cohorts(**Table 4**). Additionally, formal interaction tests were conducted within the fully adjusted model (Model 3) to statistically quantify the observed heterogeneity.

**Table 4:** Stratified analysis of the association between sdLDL-C and kidney stone risk by age and gender.

Characteristic	Model 1 OR (95% CI)	Model 2 OR (95% CI)	Model 3 OR (95% CI)	P for interaction*
China data				
Stratified by age(years)				0.09
20-39	0.75 (0.35, 1.61)	0.66 (0.30, 1.47)	0.59 (0.24, 1.45)	
40-59	0.56 (0.37, 0.85)	0.54 (0.36, 0.82)	0.46 (0.29, 0.72)	
60-85	0.52 (0.27, 1.03)	0.55 (0.27, 1.09)	0.46 (0.21, 0.98)	
Stratified by gender				0.09
Male	0.61 (0.40, 0.92)	0.59 (0.39, 0.90)	0.52 (0.33, 0.81)	
Female	0.56 (0.34, 0.91)	0.54 (0.32, 0.89)	0.43 (0.25, 0.76)	
Stratified by hypertension				0.02
No	0.51 (0.34, 0.74)	0.47 (0.32, 0.70)	0.41 (0.27, 0.63)	
Yes	0.84 (0.48, 1.47)	0.83 (0.47, 1.46)	0.67 (0.36, 1.23)	
Stratified by diabetes				0.41
No	0.55 (0.39, 0.78)	0.53 (0.37, 0.76)	0.51 (0.35, 0.75)	
Yes	0.72 (0.34, 1.53)	0.70 (0.33, 1.49)	0.60 (0.25, 1.48)	
NHANES data				
Stratified by age(years)				0.23
20-39	0.51 (0.28, 0.93)	0.49 (0.25, 0.96)	0.60 (0.31, 1.18)	
40-59	0.57 (0.36, 0.92)	0.88 (0.53, 1.46)	0.82 (0.49, 1.37)	
60-85	0.43 (0.29, 0.64)	0.59 (0.39, 0.88)	0.56 (0.37, 0.86)	
Stratified by gender				0.34
Male	0.54 (0.37, 0.79)	0.73 (0.49, 1.09)	0.73 (0.48, 1.09)	
Female	0.41 (0.28, 0.61)	0.56 (0.38, 0.85)	0.65 (0.42, 1.01)	
Stratified by hypertension				0.20
No	0.56 (0.38, 0.82)	0.62 (0.42, 0.93)	0.64 (0.42, 0.97)	
Yes	0.46 (0.32, 0.67)	0.76 (0.50, 1.14)	0.75 (0.49, 1.15)	
Stratified by diabetes				0.51
No	0.35 (0.18, 0.65)	0.38 (0.20, 0.72)	0.34 (0.17, 0.67)	
Yes	0.54 (0.40, 0.73)	0.79 (0.57, 1.08)	0.78 (0.56, 1.09)	

Model 1= No covariates were adjusted.

Model 2= Model 1+ age, gender, BMI.

Model 3 was adjusted for all covariates in **Tables 1**, **2**.

*Tested for interactivity solely within the Model 3 polished context.

#### 3.4.1 Stratification by age

In the Chinese cohort, the protective effect of sdLDL-C showed heterogeneity across age groups. The effect was most pronounced and consistent among middle-aged individuals (aged 40–59 years), with an OR of 0.46 in the fully adjusted Model 3 (95% CI: 0.29–0.72). In the elderly group (aged 60–85 years), the protective effect also reached statistical significance in Model 3 (OR = 0.46, 95% CI: 0.21–0.98). However, among younger participants (aged 20–39 years), although the point estimate (OR < 1) suggested a protective trend, the association did not achieve statistical significance in any model.

In the NHANES cohort, the protective effect was significant in the 20–39-year-old group and the 60–85-year-old group, while it lost significance in the 40–59-year-old group. However, the formal interaction tests revealed a P for interaction of 0.09 in the Chinese cohort and 0.23 in the NHANES cohort. Because these P-values are > 0.05, it indicates that the modifying effect of age is not statistically significant, and the protective trend of sdLDL-C is generally consistent across all age groups.

#### 3.4.2 Stratification by gender

In the Chinese cohort, the protective effect of sdLDL-C was consistently observed in both males and females, remaining highly significant after full adjustment in Model 3 (Males: OR = 0.52, 95% CI: 0.33–0.81; Females: OR = 0.43, 95% CI: 0.25–0.76).

In the NHANES cohort, gender stratification revealed certain differences. The protective effect was more pronounced in females, though it weakened slightly to borderline significance in Model 3 (OR = 0.65, 95% CI: 0.42–1.01). In males, the initially significant association lost statistical significance in both Model 2 and Model 3 after adjusting for covariates including age (Model 3: OR = 0.73, 95% CI: 0.48–1.09).Similar to the age stratification, the interaction tests for gender yielded a P for interaction of 0.09 and 0.34 in the Chinese and NHANES cohorts, respectively. The lack of statistical significance (P > 0.05) confirms that the association between sdLDL-C and kidney stone risk does not significantly differ between sexes.

#### 3.4.3 Stratification by comorbidity status (hypertension and diabetes)

Given the profound systemic impact of metabolic comorbidities, we further stratified the analyzes by hypertension and diabetes status (**Table 4**). Interestingly, in the Chinese cohort, a significant interactive effect was observed for hypertension (P for interaction = 0.02). The inverse association between sdLDL-C and kidney stone risk was notably more pronounced and statistically significant in patients without hypertension (OR = 0.41, 95% CI: 0.27–0.63), whereas it was attenuated in hypertensive individuals (OR = 0.67, 95% CI: 0.36–1.23). The interaction for diabetes in the Chinese cohort was not statistically significant (P for interaction = 0.41). In the NHANES cohort, the formal interaction tests revealed no statistically significant interactions for either hypertension (P for interaction = 0.20) or diabetes (P for interaction = 0.51), indicating that the protective association of sdLDL-C does not significantly differ by these comorbidity statuses in this population. However, similar to the Chinese cohort, the point estimates generally suggested a stronger protective trend in individuals without these metabolic conditions.

### 3.5 Analysis of dose response and threshold effect of sdLDL-C on kidney stone prevalence

**Figure 2:**
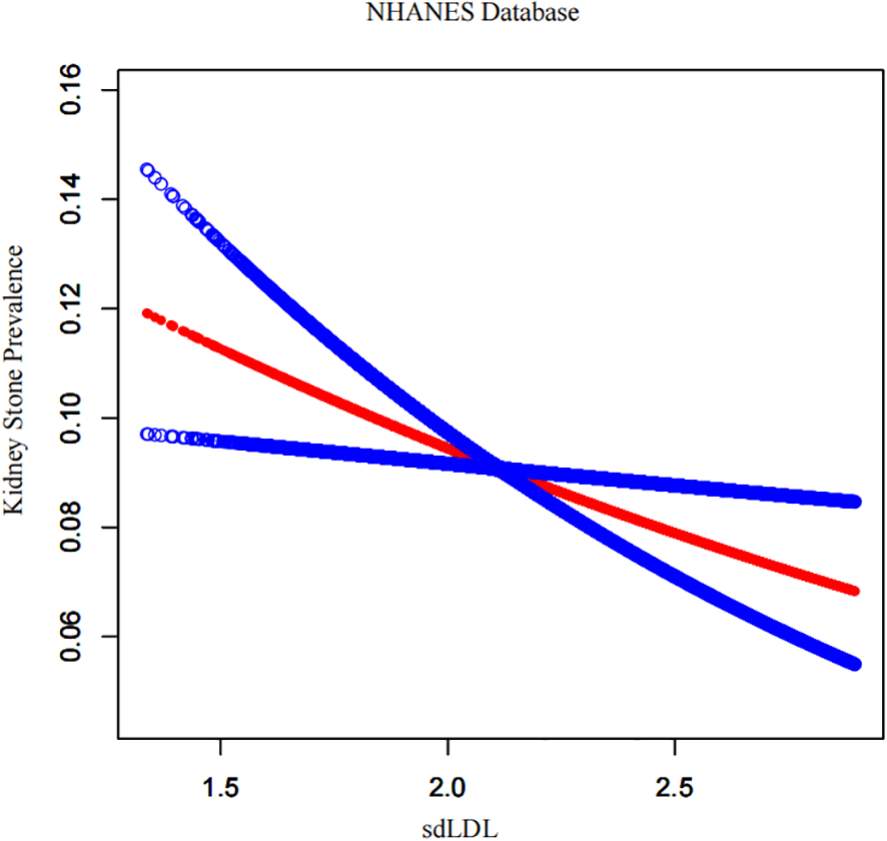
Dose–response relationship between sdLDL-C and kidney stone prevalence in the NHANES cohort.

**Figure 3:**
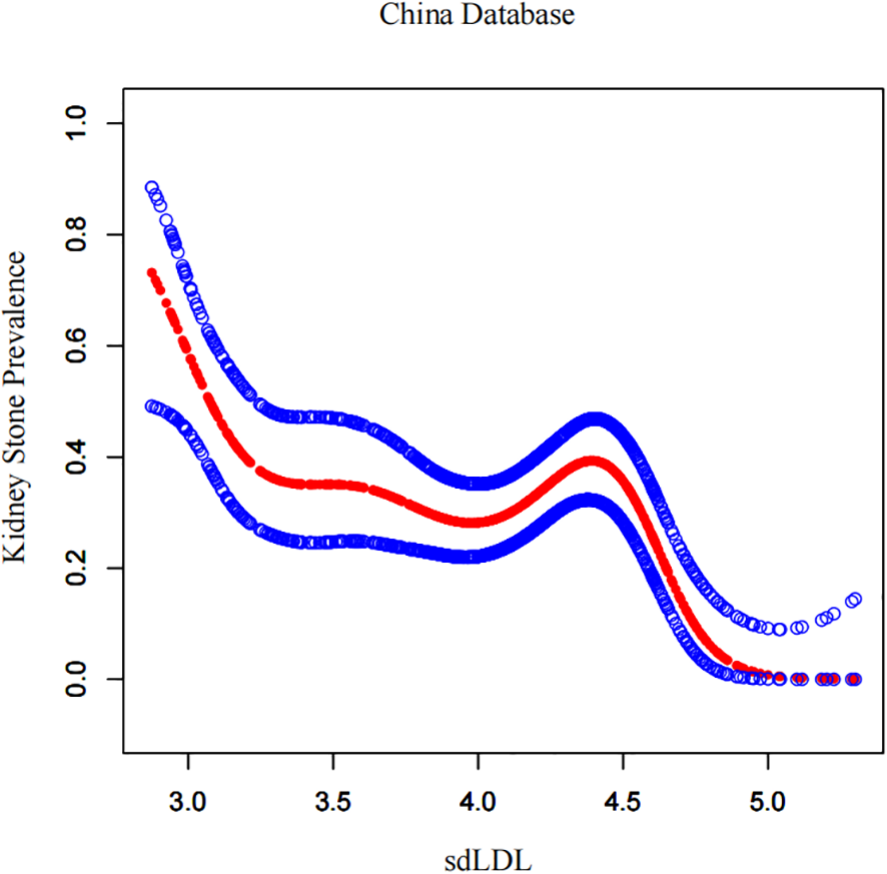
Dose–response relationship between sdLDL-C and kidney stone prevalence in the Chinese single-center cohort.

By using a generalized additive model and smoothed curve fitting, the association between the sdLDL-C index and kidney stone prevalence was further investigated. Our findings demonstrated an inverse linear relationship between the sdLDL-C index and kidney stone prevalence in NHANES database (**Figure 2**). Similarly, **Figure 3** illustrates the dose-response relationship in the single-center Chinese cohort. To rigorously evaluate whether a specific non-linear threshold existed, a threshold effect analysis utilizing a two-piecewise linear regression model was performed. However, the logarithmic likelihood ratio test revealed no significant difference between the one-line model and the two-piecewise model (P > 0.05), indicating the absence of a statistically significant inflection point. These findings suggest that the protective association of elevated sdLDL-C against kidney stone risk does not rely on a specific threshold but operates in a continuous, steady linear dose-response manner.

## 4 Discussion

By integrating a Chinese single-center cohort with data from the US NHANES, this study is the first to systematically validate a consistent inverse association between serum sdLDL-C and kidney stone risk in two independent samples with distinct population heterogeneity. This association remained significant after multiple adjustments for confounding factors, indicating that the complex role of lipoprotein metabolism in kidney stone formation may warrant re-evaluation. Notably, this protective association stands in sharp contrast to the traditional view of sdLDL-C as a highly pro-atherogenic lipoprotein (20). Such seemingly paradoxical findings are not uncommon in biomedical research and underscore that biomarkers may exert pleiotropic effects under different pathophysiological conditions. Our results suggest the need to move beyond the conventional “harmful lipid” paradigm and explore the unique interactions between lipid metabolism and the urinary microenvironment from a more systemic perspective. These empirical findings provide robust comparative data to support our hypothesis: total LDL-C is likely to crude an indicator. Because LDL particles are highly heterogeneous, the opposing biological effects of its internal subfractions may effectively cancel each other out in aggregate analyzes. Furthermore, our comorbidity-stratified analysis revealed a highly compelling phenomenon: the protective association of sdLDL-C against kidney stones was significantly modulated by the presence of hypertension in the Chinese cohort (P for interaction = 0.02). Specifically, the inverse association was highly pronounced in normotensive individuals but substantially attenuated in hypertensive patients. A plausible mechanistic explanation for this heterogeneity is that the profound systemic impact of hypertension—characterized by severe endothelial dysfunction, elevated oxidative stress, and altered renal hemodynamics—might overwhelm or mask the subtle protective mechanisms of specific lipid subfractions like sdLDL-C. In advanced metabolic states, the robust lithogenic pathways driven by hypertensive renal injury may dominate the pathogenesis of nephrolithiasis. This finding highlights the complex, competing interplay between systemic metabolic syndrome components and lipid profiles, suggesting that the clinical utility of sdLDL-C as a protective marker might be most relevant in early or relatively metabolically healthy populations.

Several plausible hypotheses may explain this inverse association and deserve further mechanistic investigation. The first hypothesis concerns direct inhibition of crystal formation, proposing that sdLDL-C particles or their surface-bound apolipoproteins may interfere with the nucleation and growth of calcium oxalate or calcium phosphate crystals in renal tubules or urine. Experimental studies have shown that certain apolipoproteins can modulate crystal formation; for example, apolipoprotein A has been identified as a biomarker for calcium oxalate stone detection (21). The second hypothesis stems from a systemic metabolic integration perspective. Kidney stones are widely recognized as a metabolic disease closely associated with insulin resistance, obesity, and metabolic syndrome (22, 23). Although sdLDL-C is a characteristic lipoprotein in insulin-resistant states (24), it may function as an integrative biomarker reflecting a specific metabolic profile in which certain metabolites or activated signaling pathways unexpectedly inhibit renal crystal formation. The third hypothesis involves the gut–kidney axis. Increasing evidence indicates that the gut microbiota influences kidney stone risk through multiple mechanisms (25), including oxalate metabolism (26). sdLDL-C levels may indirectly correspond to a distinct gut microbiota composition (27) that affects lipid metabolism and reduces stone risk through pathways such as microbial oxalate degradation (28).

Placing our findings in the context of existing literature clarifies several conflicting results from previous studies. Early observational studies generally reported a positive correlation between traditional lipid parameters and kidney stone risk (14, 29), but most did not perform LDL-C subfraction analyzes. Notably, a large-scale NHANES-based study found no independent association between calculated total LDL-C levels and stone risk (13). These seemingly inconsistent findings, combined with our results, suggest that total LDL-C may be too crude an indicator, as its internal subfractions might exert opposing effects on kidney stone risk, thereby cancelling each other out in aggregate analyzes.

Further analysis revealed significant heterogeneity in the protective effect of sdLDL-C across different populations and subgroups, highlighting findings of substantial scientific interest. In the Chinese population, the protective effect was more prominent in individuals aged over 40 years, whereas in the NHANES population, this association was more evident among young (20–39 years) and elderly (60–85 years) individuals. This cross-population difference may result from multiple interacting factors: genetic variations influencing LDL particle composition and metabolic pathways; environmental factors, particularly dietary patterns, significantly altering the metabolic context in which sdLDL-C functions (30); and differing comorbidity distributions, such as the higher prevalence of diabetes in the Chinese population (31), which may modify the association between sdLDL-C (32) and stone risk through changes in systemic metabolic status. The observed heterogeneity across genders is equally noteworthy. In the Chinese cohort, the protective effect was relatively consistent between males and females, whereas females appeared to benefit more in the NHANES population. This pattern could be explained by the dual regulatory roles of sex hormones in lipid metabolism and renal physiology. Estrogen has been shown to regulate LDL receptor activity (33) and may influence renal handling of crystalline substances (34). The observed complexity and variability in these relationships clearly highlight that demographic characteristics such as age, gender, and ethnicity represent essential modifiers of the association between sdLDL-C and kidney stone risk. These findings emphasize that future predictive models should systematically incorporate these demographic variables for improved accuracy. From a clinical management perspective, while identifying novel metabolic biomarkers like sdLDL-C is crucial, the cornerstone of kidney stone prevention and treatment remains lifestyle modification. As comprehensively highlighted in recent literature, tailored dietary adjustments and adequate water intake play an indispensable role in altering urinary supersaturation and preventing stone recurrences (35). Therefore, future clinical strategies should integrate both metabolic lipid management and rigorous dietary/hydration counseling to optimize patient outcomes.

Despite significant strengths, such as the dual-cohort validation approach, substantial sample size, and rigorous statistical adjustments, this study has several limitations warranting acknowledgment. Firstly, given its cross-sectional design, the investigation cannot establish causality, and the high likelihood of reverse causation must be acknowledged. For instance, individuals diagnosed with kidney stones often receive medical advice to adopt specific dietary modifications, increase hydration, or initiate clinical treatments (particularly lipid-lowering medications such as statins) for coexisting metabolic conditions. These clinical and lifestyle interventions could directly lower serum sdLDL-C levels. Unfortunately, specific prescription records for lipid-lowering medications and detailed dietary/hydration habits, and family history of nephrolithiasis were not comprehensively available in our retrospective Chinese cohort. Although our sensitivity analysis excluding patients with major metabolic diseases (diabetes and hypertension, Model 4) still supported a robust inverse association, the residual confounding effects of these unmeasured factors cannot be completely eliminated. Second, despite the widespread validation and use of self-reported kidney stones in the NHANES database for large epidemiological surveys, misclassification bias might still exist. Furthermore, there is inherent methodological heterogeneity in the diagnostic criteria between the two cohorts—the Chinese cohort relied on rigorous clinical imaging, whereas NHANES utilized self-reported questionnaires. While this discrepancy prevents the direct comparison of absolute prevalence, the remarkably consistent observation of the sdLDL-C inverse association across both divergent diagnostic modalities and distinct ethnic populations strongly reinforces the robustness and generalizability of our core findings. Third, the sdLDL-C levels used in our study were calculated using a validated formula. Although practical and cost-effective for large populations, this calculation method may introduce measurement errors compared with direct measurement approaches. Furthermore, we lacked detailed data regarding kidney stone composition (e.g., calcium oxalate versus uric acid stones). Given that lipid metabolism and insulin resistance may differentially influence the formation of specific stone types—particularly uric acid stones—the inability to perform subgroup analyzes based on stone pathology limits the clinical specificity of our findings. Future studies incorporating stone composition analysis are warranted to elucidate whether the protective effect of sdLDL-C varies across distinct stone subtypes. Finally, the absence of 24-hour urinary biochemical parameters constitutes another important limitation, as these factors could be significant confounders. Additionally, we did not apply multiple testing corrections (e.g., Bonferroni) to our subgroup analyzes. While this approach avoids inflating Type II errors in an exploratory setting, it inherently increases the risk of false-positive findings; thus, these subgroup trends should be interpreted with appropriate caution.

## 5 Conclusion

Our study consistently demonstrated an inverse association between serum sdLDL-C levels and kidney stone risk in two independent populations. This novel finding provides a new perspective for understanding kidney stone pathogenesis, challenging the traditional views on the pathophysiological role of sdLDL-C. Importantly, our results highlight the existence of a complex connection between lipid metabolism and kidney stone formation, extending beyond current understanding. Future research should confirm the causal direction of this association through prospective cohort studies and Mendelian randomization analyzes, employing *in vitro* experiments and animal models to investigate potential molecular mechanisms, and integrating multi-omics data to comprehensively elucidate biological pathways linking lipoprotein metabolism and stone formation. Such in-depth investigations will not only advance our knowledge of kidney stone pathogenesis but may also pave the way for innovative risk prediction tools and targeted prevention strategies.

## Data Availability

The raw data supporting the conclusions of this article will be made available by the authors, without undue reservation.

## References

[1] Mao W Zhang L Sun S Wu J Zou X Zhang G . Physical activity reduces the effect of high body mass index on kidney stones in diabetes participants from the 2007–2018 NHANES cycles: a cross-sectional study. Front Public Health. (2022) 10:936552. doi: 10.3389/fpubh.2022.936552. PMID: 35844866 PMC9283863

[2] Finger M Finger E Bellucci A Malieckal DA . Medical management for the prevention of kidney stones. Postgrad Med J. (2023) 99:112–8. doi: 10.1136/postgradmedj-2021-140971. PMID: 37222048

[3] Zhang G Zou X Mao W Chen M . Heterocyclic aromatic amines and risk of kidney stones: a cross-sectional study in US adults. Front Public Health. (2022) 10:935739. doi: 10.3389/fpubh.2022.935739. PMID: 35910865 PMC9330616

[4] Rule AD Lieske JC Li X Melton LJ Krambeck AE Bergstralh EJ . The ROKS nomogram for predicting a second symptomatic stone episode. J Am Soc Nephrol. (2014) 25:2878–86. doi: 10.1681/ASN.2013091011. PMID: 25104803 PMC4243346

[5] Ferraro PM Curhan GC D'Addessi A Gambaro G . Risk of recurrence of idiopathic calcium kidney stones: analysis of data from the literature. J Nephrol. (2017) 30:227–33. doi: 10.1007/s40620-016-0283-8. PMID: 26969574

[6] Siener R . Nutrition and kidney stone disease. Nutrients. (2021) 13. doi: 10.3390/nu13061917. PMID: 34204863 PMC8229448

[7] Akram M Idrees M . Progress and prospects in the management of kidney stones and developments in phyto-therapeutic modalities. Int J Immunopathol Pharmacol. (2019) 33:2058738419848220. doi: 10.1177/2058738419848220. PMID: 31046493 PMC6501498

[8] Hyams ES Matlaga BR . Economic impact of urinary stones. Transl Androl Urol. (2014) 3:278–83. doi: 10.3978/j.issn.2223-4683.2014.07.02. PMID: 26816777 PMC4708578

[9] Ma M Chen Y Huang H . Erythrocyte oxidative stress in patients with calcium oxalate stones correlates with stone size and renal tubular damage. Urology. (2014) 83:510.e9–510.e17. doi: 10.1016/j.urology.2013.09.050. PMID: 24360074

[10] Reiner AP Kahn A Eisner BH Pletcher MJ Sadetsky N Williams OD . Kidney stones and subclinical atherosclerosis in young adults: the CARDIA study. J Urol. (2011) 185:920–5. doi: 10.1016/j.juro.2010.10.086. PMID: 21251678 PMC3827917

[11] Hsi RS Spieker AJ Stoller ML Jacobs DR Reiner AP McClelland RL . Coronary artery calcium score and association with recurrent nephrolithiasis: the Multi-Ethnic Study of Atherosclerosis. J Urol. (2016) 195:971–6. doi: 10.1016/j.juro.2015.10.001. PMID: 26454103 PMC4966606

[12] Torricelli FC De SK Gebreselassie S Li I Sarkissian C Monga M . Dyslipidemia and kidney stone risk. J Urol. (2014) 191:667–72. doi: 10.1016/j.juro.2013.09.022. PMID: 24055417

[13] Gao M Liu M Zhu Z Chen H . The association of dyslipidemia with kidney stone: result from the NHANES 2007-2020. Int Urol Nephrol. (2024) 56:35–44. doi: 10.1007/s11255-023-03784-x. PMID: 37725273

[14] Hung J Li C Geng J Wu D Chen S . Dyslipidemia increases the risk of incident kidney stone disease in a large Taiwanese population follow-up study. Nutrients. (2022) 14. doi: 10.3390/nu14071339. PMID: 35405952 PMC9000795

[15] Toledo FGS Sniderman AD Kelley DE . Influence of hepatic steatosis (fatty liver) on severity and composition of dyslipidemia in type 2 diabetes. Diabetes Care. (2006) 29:1845–50. doi: 10.2337/dc06-0455. PMID: 16873790

[16] Fan J Liu Y Yin S Chen N Bai X Ke Q . Small dense LDL cholesterol is associated with metabolic syndrome traits independently of obesity and inflammation. Nutr Metab (Lond). (2019) 16:7. doi: 10.1186/s12986-019-0334-y. PMID: 30679939 PMC6341753

[17] Nikolic D Katsiki N Montalto G Isenovic ER Mikhailidis DP Rizzo M . Lipoprotein subfractions in metabolic syndrome and obesity: clinical significance and therapeutic approaches. Nutrients. (2013) 5:928–48. doi: 10.3390/nu5030928. PMID: 23507795 PMC3705327

[18] Sampson M Wolska A Warnick R Lucero D Remaley AT . A new equation based on the standard lipid panel for calculating small dense low-density lipoprotein-cholesterol and its use as a risk-enhancer test. Clin Chem. (2021) 67:987–97. doi: 10.1093/clinchem/hvab048. PMID: 33876239 PMC8260186

[19] Di X Liu S Xiang L Jin X . Association between the systemic immune-inflammation index and kidney stone: a cross-sectional study of NHANES 2007-2018. Front Immunol. (2023) 14:1116224. doi: 10.3389/fimmu.2023.1116224. PMID: 36895572 PMC9989007

[20] Duran EK Aday AW Cook NR Buring JE Ridker PM Pradhan AD . Triglyceride-rich lipoprotein cholesterol, small dense LDL cholesterol, and incident cardiovascular disease. J Am Coll Cardiol. (2020) 75:2122–35. doi: 10.1016/j.jacc.2020.02.059. PMID: 32354380 PMC8064770

[21] Zhu W Liu M Wang G Peng B Yan Y Che J . Fibrinogen alpha chain precursor and apolipoprotein A-I in urine as biomarkers for noninvasive diagnosis of calcium oxalate nephrolithiasis: a proteomics study. BioMed Res Int. (2014) 2014:415651. doi: 10.1155/2014/415651. PMID: 25147800 PMC4132411

[22] Shen X Chen Y Chen Y Liang H Li G Hao Z . Is the METS-IR index a potential new biomarker for kidney stone development. Front Endocrinol (Lausanne). (2022) 13:914812. doi: 10.3389/fendo.2022.914812. PMID: 35909543 PMC9329808

[23] Wong Y Cook P Roderick P Somani BK . Metabolic syndrome and kidney stone disease: a systematic review of literature. J Endourol. (2016) 30:246–53. doi: 10.1089/end.2015.0567. PMID: 26576717

[24] Kim S Lee J Lee Y Song Y Linton JA . Association between triglyceride-glucose index and low-density lipoprotein particle size in Korean obese adults. Lipids Health Dis. (2023) 22:94. doi: 10.1186/s12944-023-01857-5. PMID: 37403101 PMC10318677

[25] Ticinesi A Nouvenne A Meschi T . Gut microbiome and kidney stone disease: not just an Oxalobacter story. Kidney Int. (2019) 96:25–7. doi: 10.1016/j.kint.2019.03.020. PMID: 31229040

[26] Suryavanshi M Franklin A Assimos DG Knight J Miller AW . Baseline abundance of oxalate-degrading bacteria determines response to Oxalobacter formigenes probiotic therapy. Gut Microbes. (2025) 17:2562337. doi: 10.1080/19490976.2025.2562337. PMID: 40984794 PMC12459359

[27] Chao Z Xin L Xiaoling D Wenbo X Tingting S Di H . The effects of Bifidobacterium animalis subsp. lactis BLa80 on glycemic control and gut microbiota in patients with T2DM: a randomized, double-blind, placebo-controlled trial. J Diabetes Complications. (2025) 39:109195. doi: 10.1016/j.jdiacomp.2025.109195. PMID: 41110348

[28] Noonin C Thongboonkerd V . Beneficial roles of gastrointestinal and urinary microbiomes in kidney stone prevention via their oxalate-degrading ability and beyond. Microbiol Res. (2024) 282:127663. doi: 10.1016/j.micres.2024.127663. PMID: 38422861

[29] Liu C Ho K Tsai Y Huang H . Increased renal uptake and urine excretion of oxidized LDL is possibly associated with formation of large calcium oxalate nephrolithiasis: a preliminary study. World J Urol. (2023) 41:1423–30. doi: 10.1007/s00345-023-04360-9. PMID: 36977786

[30] Santos HO Earnest CP Tinsley GM Izidoro LFM Macedo RCO . Small dense low-density lipoprotein-cholesterol (sdLDL-C): analysis, effects on cardiovascular endpoints and dietary strategies. Prog Cardiovasc Dis. (2020) 63:503–9. doi: 10.1016/j.pcad.2020.04.009. PMID: 32353373

[31] Yang L Shao J Bian Y Wu H Shi L Zeng L . Prevalence of type 2 diabetes mellitus among inland residents in China (2000-2014): a meta-analysis. J Diabetes Investig. (2016) 7:845–52. doi: 10.1111/jdi.12514. PMID: 27181391 PMC5089946

[32] He Q Wang L Fang Y Liang M Chen X Hu R . Relationship of small dense low-density lipoprotein cholesterol level with pre-diabetes and newly detected type 2 diabetes. Sci Rep. (2025) 15:19500. doi: 10.1038/s41598-025-03133-1. PMID: 40461580 PMC12134114

[33] Zhu X Bonet B Gillenwater H Knopp RH . Opposing effects of estrogen and progestins on LDL oxidation and vascular wall cytotoxicity: implications for atherogenesis. Proc Soc Exp Biol Med. (1999) 222:214–21. doi: 10.1046/j.1525-1373.1999.d01-138.x. PMID: 10601880

[34] Peerapen P Thongboonkerd V . Protective cellular mechanism of estrogen against kidney stone formation: a proteomics approach and functional validation. Proteomics. (2019) 19:e1900095. doi: 10.1002/pmic.201900095. PMID: 31475403

[35] Rossi M Barone B Di Domenico D Esposito R Fabozzi A D'Errico G . Correlation between ion composition of oligomineral water and calcium oxalate crystal formation. Crystals. (2021) 11:1507. doi: 10.3390/cryst11121507. PMID: 41725453

